# Vimentin on the move: new developments in cell migration

**DOI:** 10.12688/f1000research.15967.1

**Published:** 2018-11-15

**Authors:** Rachel A. Battaglia, Samed Delic, Harald Herrmann, Natasha T. Snider

**Affiliations:** 1 Department of Cell Biology and Physiology, University of North Carolina, Chapel Hill, NC, USA; 2 Division of Molecular Genetics, German Cancer Research Center, Heidelberg, Germany; 3 Institute of Neuropathology, University Hospital Erlangen, Erlangen, Germany

**Keywords:** cytoskeleton, cell migration, cell polarity, cell stiffness, post-translational modifications

## Abstract

The vimentin gene ( *VIM*) encodes one of the 71 human intermediate filament (IF) proteins, which are the building blocks of highly ordered, dynamic, and cell type-specific fiber networks. Vimentin is a multi-functional 466 amino acid protein with a high degree of evolutionary conservation among vertebrates. *Vim ^−/−^* mice, though viable, exhibit systemic defects related to development and wound repair, which may have implications for understanding human disease pathogenesis. Vimentin IFs are required for the plasticity of mesenchymal cells under normal physiological conditions and for the migration of cancer cells that have undergone epithelial–mesenchymal transition. Although it was observed years ago that vimentin promotes cell migration, the molecular mechanisms were not completely understood. Recent advances in microscopic techniques, combined with computational image analysis, have helped illuminate vimentin dynamics and function in migrating cells on a precise scale. This review includes a brief historical account of early studies that unveiled vimentin as a unique component of the cell cytoskeleton followed by an overview of the physiological vimentin functions documented in studies on *Vim ^−/−^* mice. The primary focus of the discussion is on novel mechanisms related to how vimentin coordinates cell migration. The current hypothesis is that vimentin promotes cell migration by integrating mechanical input from the environment and modulating the dynamics of microtubules and the actomyosin network. These new findings undoubtedly will open up multiple avenues to study the broader function of vimentin and other IF proteins in cell biology and will lead to critical insights into the relevance of different vimentin levels for the invasive behaviors of metastatic cancer cells.

## Introduction

Vimentin is an intermediate filament (IF) protein whose name is derived from the Latin word *vimentum*, which means wickerwork ^1^. Early observations with immunofluorescence microscopy revealed a complex fiber network, distinct from the already-known keratin system in the cytoskeleton of epithelial cells ^1^. In mouse development, vimentin initially emerges in a highly migratory cell type (that is, when the embryo is still a two-layered epithelium and ectodermal cells start to migrate into the newly forming “mesodermal cleft”). In these first mesenchymal cells, keratin genes are turned off and the vimentin gene is turned on ^2^. Postnatal expression of vimentin is restricted to fibroblasts, endothelial cells, lymphocytes, and several specialized cells of the thymus and the brain ^3, 4^. Moreover, it was observed early on that vimentin is significantly expressed in most cell types, particularly tumor cells, when the cells are taken from primary tissues and transitioned into culture ^5^. The mechanism behind this widespread expression of vimentin is a serum response element in the *VIM* promoter, which responds to factors present in the serum that culture media are complemented with ^6, 7^. Therefore, many cell types expressing vimentin in culture are not ideal models to study the genuine biological functions of vimentin. However, with appropriate cell systems, it has been demonstrated that vimentin plays an important role in various physiological situations. For instance, upregulation of vimentin in cultured epithelial cells ^8, 9^ and *in vivo* ^10^ correlates with epithelial–mesenchymal transition (EMT), a process that occurs during development, wound healing, and cancer metastasis ^11^. Though originally described as a “skeletal” element of cells, the vimentin filament network was revealed by live-cell imaging studies to be a very dynamic system ^12^. Specifically, FRAP (fluorescence recovery after photobleaching) studies demonstrated that vimentin in interphase BHK-21 cells had a recovery half-time of 5 ± 3 minutes ^12^, exhibiting dynamic properties similar to those of microtubules ^13^ and actin filaments ^14^.

Small molecules for the selective targeting of vimentin (and other IFs) are currently not available, which has limited mechanistic understanding of this cytoskeletal component. The first global vimentin knockout mouse was generated 25 years ago and described as having no phenotype ^15^, which was frequently and mistakenly taken as evidence that, despite its extreme evolutionary conservation in vertebrates ^16^, vimentin is of little physiological importance. While early embryogenesis and litter size are unaffected in the *Vim ^−/−^* mice, a number of phenotypes reported in the literature support multiple functions of vimentin at the cellular level in the maintenance of stemness ^17^, differentiation ^18, 19^, proliferation ^18^, adhesion ^20^, migration ^21, 22^, and invasion ^23^. The cellular-level defects in the *Vim ^−/−^* mice cause impairments in normal physiological processes, such as mammary gland development ^17^, angiogenesis ^24^, vascular stiffness ^25^, steroidogenesis ^26^, glia development ^27^, and myelination of peripheral nerves ^28^. Of particular relevance to human disease pathogenesis, *Vim ^−/−^* mice have defects in wound healing and exhibit differences in tissue repair after injury to the skin ^18^, eye ^29, 30^, brain ^31– 33^, vasculature ^34, 35^, lung ^36, 37^, kidney ^10, 38, 39^, and gut ^40, 41^. According to studies using the global *Vim ^−/−^* mice, the “true” function of vimentin is at the organismal level of cells and is important under both physiological and pathophysiological stress conditions.

There are no known monoallelic diseases resulting from missense mutations in vimentin, in contrast to other IF genes. In general, disease-causing mutations are less likely to occur in genes with extensive molecular interaction networks compared with genes with more restricted connectivities ^42^. Currently, the number of unique interactions documented for vimentin in the Biological General Repository for Interaction Datasets is 276, which is severalfold higher than that for IF genes with known disease-causing mutations, including *KRT5* (66), *KRT14* (45), *DES* (47), *GFAP* (95), and *NEFL* (52) ^43^ ( https://thebiogrid.org). This view of vimentin functioning within a large molecular network is supported by studies showing that dominant negative vimentin mutations that disrupt filament formation interfere with cellular proteostasis pathways and apoptosis ^44^ and are associated with the development of cataracts in mice ^45^ and humans ^46^. With these historical facts in mind, we will review new findings relevant to the role of vimentin in migratory processes of cells and tissues.

## Novel roles of vimentin in cell migration

### Vimentin promotes the migration of different cell types

It is well appreciated that motile and invasive cells express higher levels of vimentin ^47, 48^ and that vimentin knockout or knockdown attenuates the migration of fibroblasts ^48, 49^, leukocytes ^20^, astrocytes ^50^, and various cancer cell types ^8, 51, 52^. For a broader overview of the functions of vimentin and other IFs in cell biology ^53^ (and cell migration in particular), we refer the readers to previous reviews ^54– 57^. Here, we specifically focus on the most recent studies illuminating how vimentin orchestrates cytoskeletal rearrangements and mechano-signaling to promote cell migration. In particular, we will discuss how the flexibility of the vimentin scaffold is modulated to provide a plastic “net” dynamically enforcing the rigid actomyosin motor system.

### Vimentin filaments pattern microtubules during directed migration

Establishment of persistent cell polarity is a key property of migrating cells responding to internal and external signals that guide directionality of movement ^58^. The high turnover rate of the microtubule network, which occurs in the order of 3 to 5 minutes, stabilizes cell polarity during directed cell migration ^59, 60^. The vimentin filament network is closely associated with, and functionally dependent on, microtubules ^61– 63^ and microtubule-associated molecular motors ^64, 65^. This is reflected in the drastic vimentin reorganization, often as an apparent “collapse” around the cell nucleus, upon disrupting microtubules with colchicine ^62^. Recent work by Gan *et al*. used a systematic quantitative approach to characterize the co-dependent behavior of these two cytoskeletal systems during cell migration ^66^. The authors subjected a retinal pigment epithelium (RPE) cell line expressing fluorescently tagged vimentin and tubulin (under the control of their endogenous promoters) to a scratch wound assay in a confluent monolayer followed by live-cell imaging and computational image analysis ^66^. The study revealed that vimentin filaments are stable for up to 20 minutes after nocodazole treatment and that microtubules that associated with vimentin were more resistant to nocodazole treatment. Furthermore, microtubules used vimentin filaments as a growth template after nocodazole washout. The major conclusion from this work is that, during directed cell migration, long-lived vimentin filaments guide the growth of microtubule plus ends along the preceding microtubule tracks, thus providing a form of “memory” required for the continuous maintenance of cell polarity by the short-lived microtubules ^66^. The molecular nature of the microtubule–vimentin interaction is not clear at present, although there is strong evidence that it is mediated via other proteins, such as adenomatous polyposis coli (APC) ^67^, which links vimentin to microtubules, or it may be mediated via post-translational modifications (PTMs) ^68^, such as phosphorylation. Additionally, it remains to be determined whether, and how, this mechanism applies to cells migrating *in vivo*, since RPE cells *in vivo* express keratins but lack vimentin ^69^.

### Vimentin regulates cell migration by restricting actin flow and aligning traction stress

Cell migration is dependent on actin filaments, which reorganize into different arrays to support the formation of membrane protrusions (for example, lamellipodia and filopodia) and propel the cell along its substrate ^70^. Vimentin interacts with actin filaments directly via its tail domain ^71^ and indirectly via the cytolinker protein plectin ^72^. Another vimentin binding partner, the capping protein (CP) regulator CARMIL2 (CP, Arp2/3, myosin-I linker 2), facilitates lamellipodia formation and cell migration in a vimentin-dependent manner ^73^.

Jiu *et al*. showed recently that transverse arcs, which are actin bundles containing the motor protein myosin II, are essential for the retrograde flow of small vimentin particles and their incorporation into perinuclear vimentin filaments in the osteosarcoma U2OS cells ^74^. The small vimentin particles, called *squiggles* ^75^, represent intermediates of synthesis-independent filament turnover that occurs through severing and re-annealing ^76^ or subunit exchange ^77^. Upon depletion of transverse arcs by knockdown of tropomyosin 4, which recruits myosin II ^78^, retrograde flow and perinuclear localization of vimentin were lost ^74^. While vimentin deficiency did not affect stress fiber formation in this particular system, it caused transverse arcs to pull away from the leading edge, suggesting that vimentin controls actin dynamics by restricting the retrograde flow of transverse arcs ^74^. The cross-talk between vimentin and actin transverse arcs is dependent on plectin ^74^ and appears to be important for nuclear positioning, a key element of cell migration, and other processes ^79^. To that end, vimentin was recently shown to interact with the nuclear pore complex protein Nup88 ^80^, which may bear consequences to nuclear positioning during cancer cell migration ^81^.

**Figure 1.**
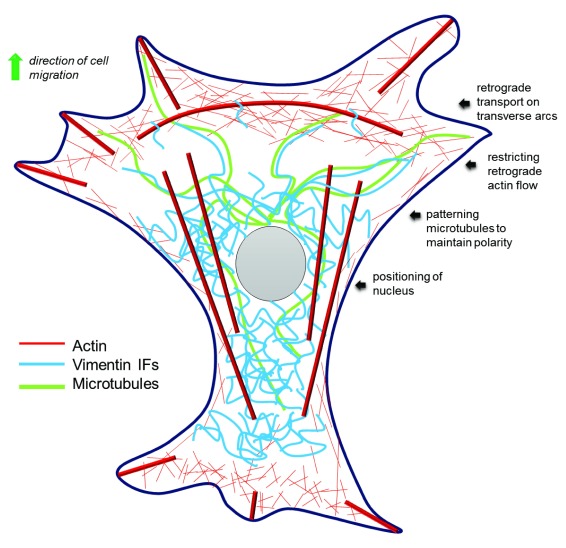
Vimentin promotes directed cell migration by coordinating the dynamics of actin filaments and microtubules. Vimentin particles disassemble at the cell periphery and undergo retrograde transport on transverse actin arcs for likely incorporation into mature filaments. Mature vimentin filaments restrict retrograde actin flow and control nuclear positioning during cell migration. Long-lived vimentin filaments coordinate the maintenance of cell polarity during migration by closely associating with relatively short-lived microtubules to pattern the growth of new microtubules along their preceding tracks. IF, intermediate filament.

Because vimentin filaments are highly dynamic, a key question is how the various physical states of the network control cellular behavior. For example, phosphorylation-dependent vimentin disassembly at the cell periphery is required for the formation of actin-based lamellipodia membrane protrusions ^49^. The Danuser group developed a novel computational method to analyze vimentin filaments and showed that long (>4 µm) vimentin fibers serve as a load-bearing scaffold to buffer traction stress during single-cell migration ^82^. Using traction force microscopy on non-immortalized human skin fibroblasts, the authors observed that actin moved 14 times faster in areas devoid of vimentin and two times faster in areas containing a rarefied vimentin “mesh” when compared with areas of the cell that were occupied by fibrous vimentin ^82^. Thus, these findings align with the work by Jiu *et al*. ^74^ with respect to the function of filamentous vimentin in restraining retrograde actin flow. Additionally, traction forces distributed non-specifically throughout the interface between the cell and the substrate in vimentin-deficient cells while, in the presence of vimentin, actomyosin forces were redirected to peripheral adhesions ^82^. Therefore, the current understanding is that mature vimentin fibers restrict the formation of lamellipodia and actin flow while facilitating the alignment of traction forces to promote single-cell migration in collaboration with microtubules ( Figure 1).

### Vimentin promotes collective cell migration by restraining traction forces and supporting lateral cell–cell contacts

In addition to vimentin, actin transverse arcs regulate the perinuclear localization of nestin ^74^, an IF protein that cannot form filaments on its own but can co-assemble with vimentin in various cell types, such as astrocytes ^83^. Astrocytes are specialized glial cells critical for central nervous system (CNS) function ^84^. The IF cytoskeleton of astrocytes is composed of vimentin, nestin, and glial fibrillary acidic protein (GFAP) ^83, 85^. Whereas GFAP is the major IF protein of mature astrocytes under basal conditions, vimentin is highly expressed by astrocytes during normal development and in CNS injury ^85^. In developing *Xenopus laevis* embryos, vimentin-expressing cells first appear lining the forming neural tube ^86^, indicating that these cells are radial glia guiding migratory neuronal cells ^87^.

There is strong evidence that astrocyte migration is implicated in CNS development ^88, 89^, injury ^90, 91^, and glioma tumor formation ^92^. Combined reduction of the protein levels of vimentin, GFAP, and nestin decreases astrocyte speed, directionality, and persistence of movement during collective cell migration ^93^, the coordinated movement of cells as groups, in a manner dependent on cell–cell contact ^94^. In a scratch wound assay using primary rodent astrocytes, knockdown of vimentin, along with GFAP and nestin, promotes an increase in actin stress fibers perpendicular to the wound, a reduction in actin stress fibers parallel to the wound, and a reduction in retrograde actin flow ^93^. Triple IF knockdown in astrocytes additionally alters the morphology of adherens junctions (AJs) and decreases the retrograde flow of AJs measured by live imaging of N-cadherin and loss of vinculin localization to AJs ^93^. Finally, the astrocyte IF system restricted the mechanical coupling of focal adhesion to the actomyosin network. Given the interdependent nature of astrocyte IFs and the triple IF knockdown strategy used in this study, it is not possible to assign a specific role of vimentin per se. However, in light of the additional studies supporting similar roles of vimentin in other cell types, vimentin is a likely key regulator of astrocyte migration. Overall, these findings may have functional implications for gliomas, since high vimentin expression is an independent prognostic factor for their metastatic aggressiveness ^95^.

### Vimentin promotes cell migration by enhancing contact-dependent cell stiffening

Upregulation of vimentin in epithelial cells, in addition to increasing cell motility ^96^, induces physical changes in cell shape, loss of cell–cell contacts, and increased turnover of focal adhesions ^48^. Furthermore, vimentin supports cellular elasticity and protects against mechanical stress, such as compression ^97^. Tumor cells experience significant compressive stress as they grow, which is known to promote cell migration and invasion related to the formation of new leader cells and actomyosin-independent cell extensions in breast cancer cells ^98^.

Using a number of biophysical methods coupled with cell migration assays under low- and high-cell-density conditions, Messica *et al*. showed that vimentin controls cell migration in dense, but not sparse, cultures ^99^. Using the invasive breast carcinoma MDA-MB-231 cells as a model system, the authors compared how the presence or absence of vimentin regulates their mechanical, migratory, and invasive properties. Vimentin-lacking MDA-MB-231 cells were softer and more deformable ^99^, which are characteristics attributed to more invasive and metastatic cancer cells ^100^. Interestingly, the loss of vimentin significantly diminished the ability of MDA-MB-231 cells to migrate and invade in dense, but not sparse, cultures, while vimentin expression positively correlated with longer persistence time of migration ^99^. The latter is in line with the previous study supporting a role for vimentin in microtubule-dependent cell polarity regulation during migration ^66^. The authors proposed that the decreased migration and invasiveness of the “softer” vimentin-negative MDA-MB-231 cells relate to their deformability in crowded spaces, such that each cell can be molded to accommodate neighboring cells, losing its polarity in the course of this process. In the presence of vimentin, the cells are able to stiffen and redirect their migration to move toward vacant intercellular spaces. It would be intriguing to explore whether and how vimentin regulates cytoskeleton reorganization and cellular stiffening during cancer cell migration through soft substrates, as was recently reported ^101^.

## Novel regulators of vimentin

### Vimentin regulation by microRNAs

Vimentin expression is elevated in cancer development and progression, as demonstrated by multiple recent studies using *in vivo* cancer metastasis models ^23, 102, 103^. Therefore, factors that regulate vimentin expression are of particular interest. MicroRNAs (miRs) are small, non-coding RNA molecules that are involved in gene regulation by binding to the 3′ untranslated region of the target mRNA to promote degradation or prevent translation ^104^. miRs are well-recognized regulators of wound healing, EMT, and cancer metastasis ^104^. It was shown recently that vimentin expression is inhibited by miR-548an, resulting in reduced invasion and proliferation of pancreas cancer cells ^105^. Another study found that miR-22 acts as an EMT antagonist by blocking the expression of the transcription factors Snail and Slug, as well as vimentin mRNA, and by increasing the expression of E-cadherin mRNA ^106^. This study found that miR-22 is a direct inhibitor of Snail and ERK2 and that ERK2 is involved in a regulatory feedback loop with Slug and vimentin. Specifically, ERK2 activates Slug, which in turn promotes vimentin-dependent ERK2 phosphorylation and decreased apoptosis ^106^. The relationship between vimentin and miRs is bi-directional, since vimentin blocks the function of several miRs, including miRs 182, 203, 887, and 3619 ^107^, which are proposed to be tumor suppressor molecules that prevent phospholipase D-associated cancer cell migration and invasion ^107^.

### Vimentin regulation by post-translational modifications

Vimentin and other IF proteins are extensively modulated by PTMs under normal conditions and in disease settings ^108, 109^. Recent studies have revealed novel regulators of vimentin, including the E3 ubiquitin ligase TRIM56, which promotes ubiquitination and proteasome-dependent degradation of vimentin ^110^. In addition, vimentin interacts directly with ubiquilin 2 (UBQLN2) and myotubularin-1 (MTM1) as demonstrated by proteomics studies ^111^. Knockdown of either UBQLN2 or MTM1 increased vimentin protein expression and decreased proteasome activity. Vimentin is known to be glycosylated at multiple sites on the head domain ^112– 114^, and recently Tarbet *et al*. ^115^ demonstrated that glycosylation of vimentin is required for vimentin crosslinking, filament formation, and cell migration ^114^. Since glycosylation is significantly altered and functionally important in cancer development and progression ^109^, the findings from this study bear importance for understanding the role of vimentin in cancer at a mechanistic level.

## Conclusions

Vimentin is a key component of the cytoskeleton with important biological functions at the cellular and organismal levels. Vimentin is particularly important during development and in cancer during EMT and metastasis. Vimentin interacts with, and regulates, microtubules, actin, focal adhesions, and AJs during cell migration. Recent studies highlight that environmental factors, such as cell density and substrate stiffness, should be carefully considered when studying the role of vimentin in cell migration in vitro. Overexpression and tagging of vimentin can cause defects in the filament network, so novel gene editing strategies at endogenous loci should be used to determine the importance of specific vimentin residues and their respective modifications in filament dynamics. One such approach could be to focus on frequently reported PTM sites on vimentin that have been curated by the comprehensive PhosphoSitePlus database ^116^ but not validated via mechanistic studies. This approach was applied previously on keratin 8 to reveal conserved tyrosine phosphorylation as an important regulator of solubility and filament dynamics ^117^. Future development of small molecules that selectively target vimentin and control the assembly state of vimentin filaments will be essential to understand vimentin dynamics and to target its function as a means to modulate cell migration.
